# Recurrent Neural Networks with Integrated Gradients Explanation for Predicting the Hysteresis Behavior of Shape Memory Alloys

**DOI:** 10.3390/s26010110

**Published:** 2025-12-24

**Authors:** Dmytro Tymoshchuk, Oleh Yasniy, Iryna Didych, Pavlo Maruschak, Nadiia Lutsyk

**Affiliations:** 1 Department of Artificial Intelligence Systems and Data Analysis, Ternopil Ivan Puluj National Technical University, 46001 Ternopil, Ukraine; oleh.yasniy@gmail.com; 2 Department of Computer-Integrated Technologies, Ternopil Ivan Puluj National Technical University, 46001 Ternopil, Ukraine; iryna.didych1101@gmail.com; 3 Department of Automation of Technological Processes and Production, Ternopil Ivan Puluj National Technical University, 46001 Ternopil, Ukraine; 4 Department of Computer Systems and Networks, Ternopil Ivan Puluj National Technical University, 46001 Ternopil, Ukraine; lutsyk.nadiia@tntu.edu.ua

**Keywords:** SMA, hysteresis, cyclic loading, RNN, LSTM, GRU, integrated gradients, XAI, machine learning, neural networks

## Abstract

The study presents an approach to predicting the hysteresis behavior of shape memory alloys (SMAs) using recurrent neural networks, including SimpleRNN, LSTM, and GRU architectures. The experimental dataset was constructed from 100 to 250 loading–unloading cycles collected at seven loading frequencies (0.1, 0.3, 0.5, 1, 3, 5, and 10 Hz). The input features included the applied stress σ (MPa), the cycle number *N* (the Cycle parameter), and the indicator of the loading–unloading stage (UpDown). The output variable was the material strain ε (%). Data for training, validation, and testing were split according to the group-based principle using the Cycle parameter. Eighty percent of cycles were used for model training, while the remaining 20% were reserved for independent assessment of generalization performance. Additionally, 10% of the training portion was reserved for internal validation during training. Model accuracy was evaluated using MAE, MSE, MAPE, and the coefficient of determination R^2^. All architectures achieved R^2^ > 0.999 on the test sets. Generalization capability was further assessed on fully independent cycles 251, 260, 300, 350, 400, 450, and 500. Among all architectures, the LSTM network showed the highest accuracy and the most stable extrapolation, consistently reproducing hysteresis loops across frequencies 0.1–3 Hz and 10 Hz, whereas the GRU network showed the best performance at 5 Hz. Model interpretability using the Integrated Gradient (IG) method revealed that Stress is the dominant factor influencing the predicted strain, contributing the largest proportion to the overall feature importance. The UpDown parameter has a stable but secondary role, reflecting transitions between loading and unloading phases. The influence of the Cycle feature gradually increases with the cycle number, indicating the model’s ability to account for the accumulation of material fatigue effects. The obtained interpretability results confirm the physical plausibility of the model and enhance confidence in its predictions.

## 1. Introduction

Over the past decades, shape memory alloys (SMAs) have evolved from a subject of purely fundamental research into an essential functional component of modern high-technology engineering systems. SMAs are widely used in medicine [1,2], the aviation [3,4] and space [5,6] industries, robotics [7,8], automation systems [9,10], the automotive sector [11,12], and civil engineering [13,14].

**Figure 1:**
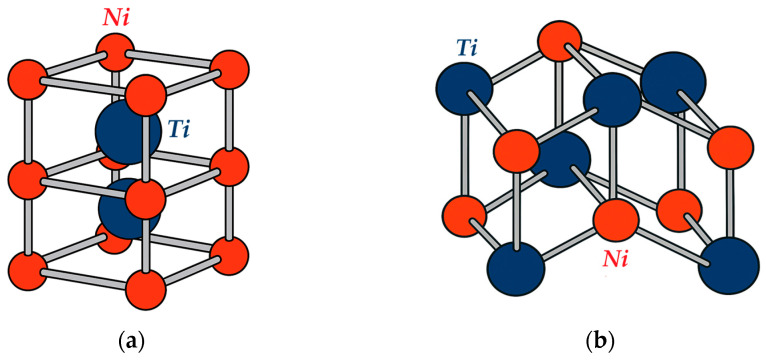
Crystal structure of NiTi (**a**) austenitic phase B2 and (**b**) martensitic phase B19′.

Among the wide variety of shape memory alloys, NiTi-based alloys have achieved the greatest industrial adoption. Historically, they have become the benchmark class of SMAs due to their combination of moderate phase-transformation temperatures, high corrosion resistance, and excellent biocompatibility. SMAs are characterized by their ability to transition between two primary crystalline states—austenite and martensite. The austenitic phase has a body-centered cubic B2 lattice (with Ni atoms positioned at the cube corners and Ti atoms at the center), which provides high stiffness and a large yield strength (Figure 1a). The martensitic phase, by contrast, is described by a monoclinic B19′ lattice in which the planes are sheared relative to the original cubic cell, imparting significant ductility to the alloy (Figure 1b).

SMAs exhibit two unique functional properties: the Shape Memory Effect (SME) and Superelasticity (SE), the latter often referred to as Pseudoelasticity (PE).

The Shape Memory Effect occurs during temperature changes, during which the alloy undergoes sequential phase transformations. Upon cooling, austenite transforms first into twinned martensite and subsequently into detwinned martensite. When heated, the material reverts to the austenitic state. This thermally driven transformation cycle repeats each time the alloy is cooled and reheated. Superelasticity, in contrast, manifests under isothermal conditions when the material temperature is equal to or exceeds the austenite finish temperature. Under these conditions, the alloy remains in a stable austenitic phase and transforms into detwinned martensite upon application of mechanical loading. Upon unloading, the structure returns to the austenitic state, fully recovering its original shape. This superelastic thermomechanical cycle begins at elevated temperatures in a fully austenitic state, proceeds through the formation of stable detwinned martensite under loading, and concludes with complete reversion to austenite upon unloading.

**Figure 2:**
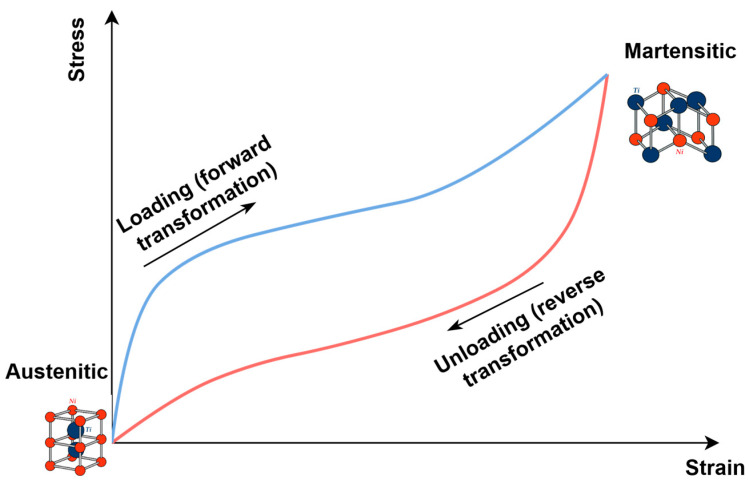
Hysteresis loop of one SMA loading and unloading cycle.

Owing to their ability to undergo reversible phase transformations, SMAs can recover their original shape in response to changes in temperature or applied mechanical loading. This unique mechanism is governed by microstructural rearrangements and the interplay between the material’s mechanical and functional properties. As a result of the reversible transformations occurring during the superelastic cycle, a hysteresis loop appears in the stress–strain space, the area of which corresponds to the energy dissipated by the material during transitions between austenite and martensite (Figure 2).

The presence of energy dissipation reflects the alloy’s ability to convert part of the mechanical energy into heat during the austenite–martensite transformation. This behavior characterizes the material’s damping capacity and its superelastic response. Owing to these properties, shape memory alloys are highly effective for vibration suppression and operation under dynamic loading conditions. The dimensions of the hysteresis loop depend on the alloy’s chemical composition, thermal treatment, and external loading conditions.

The main advantages of SMA in actuators and sensors are their simple design and high degree of miniaturization [15]. Traditionally, actuators and sensors are based on elements made of functional materials (bimetallic plates, piezoelectric elements, etc.) capable of converting electrical, thermal, and magnetic energy into mechanical energy. Compared to these types of materials, SMAs offer lower actuator response times but significantly higher specific power output. They can also serve as both a control system and an actuator [16]. These actuators and sensors can operate independently of complex drives and mechanical systems, thereby reducing the overall weight and size of the devices.

The application of machine learning methods spans a wide range of research domains, including materials science [17,18,19,20,21,22,23], medicine [24,25,26,27,28,29], finance [30,31,32,33,34], engineering [35,36,37,38,39], and information technology [40,41,42,43,44]. There are known studies that deal with neural network modeling of the stress–strain state of materials with shape memory and structures made from them, which enable a careful consideration of their features and properties, ensuring the stability of operational characteristics in the presence of instability in macro- and microstructural parameters. Studying the obtained patterns can facilitate the analysis of the correlation between SMA microstructure and hysteresis behavior [45,46].

Due to their ability to process large datasets and identify complex nonlinear relationships, machine learning techniques enable the effective prediction of system and material behavior under diverse operating conditions. A considerable research effort has focused on modeling the properties of shape memory alloys using machine learning algorithms. Numerous studies confirmed the effectiveness of such approaches for predicting key SMA characteristics. The work in [47], for example, presents a comprehensive review of artificial neural network applications for modeling SMA properties, covering various neural network architectures and their performance in reproducing SMA behavior. In [48,49], a universal approach for predicting the martensitic transformation temperature in SMAs using machine learning techniques is proposed.

The authors derive an empirical formula for the martensitic transformation temperature, demonstrating strong generalizability across various SMA types. In [50], the feasibility of predicting the loading frequency of SMAs from experimental data using different machine learning methods is investigated. The study specifically shows that a multilayer perceptron (MLP) neural network achieves the highest classification accuracy. Predicting the loading frequency from known input parameters (such as stress, strain, and cycle number) allows researchers to determine the frequency at which the material dissipates a given amount of energy, which is essential for analyzing the functional behavior of SMAs. The study [51] employs machine learning methods to expedite the design of NiTi alloys with enhanced elastocaloric effects, which is promising for solid-state cooling. Nine new alloys were discovered during the learning process. The model showed high interpretability and efficiency. In article [52], a machine learning method is proposed for identifying the thermodynamic parameters of superelastic SMAs, which considers the dependence on the deformation rate. In study [53], machine learning models were developed to predict the mechanical properties of nickel-free titanium shape memory alloys. The research focused on forecasting the ultimate tensile strength (UTS) and elongation (EL). The Gradient Boosting Regression model demonstrated the highest accuracy for predicting EL, whereas the prediction of UTS proved to be less accurate. In [54], an LSTM-based model was developed to predict the rotational angle of a pulley in an SMA-wire-driven actuator, taking into account the hysteresis behavior of the alloy. In [55], an interpretable piecewise-linear regression model was developed and experimentally validated to identify the ranges of chemical and physical properties that lead to either B19 or B19′ transformation, as well as to predict a parameter derived from the chemical characteristics of the alloy composition. In study [56], a machine learning approach was employed to identify NiTiHf alloys suitable for actuator applications in space environments. The K-nearest neighbors model achieved the highest accuracy, enabling effective prediction of phase transformation temperatures. In [57], the authors demonstrated the effectiveness of statistical learning for predicting the martensitic transformation temperature of SMAs using three descriptors related to chemical bonding and atomic radii of the alloying elements. This approach accelerated the discovery of alloys with targeted transformation temperatures. In study [58], the behavior of NiTi SMAs was analyzed using a neural-network-based modeling approach, where the model predicted strain as a function of temperature. In [59], a platform was introduced for discovering new SMA compositions exhibiting narrow temperature hysteresis loops under applied loading. The work in [60] examined the use of a deep neural network to predict the outcomes of electrochemical processing of NiTi SMA. The model outperformed traditional approaches, demonstrating lower RMSE values and higher predictive accuracy. In a study [61], a neural network model was proposed to predict temperature and displacement in SMA-based actuators, eliminating the need for bulky position sensors.

One of the key limitations of studies that employ machine learning to predict SMA properties is the insufficient volume of experimental data. The limited availability of high-quality and representative datasets hinders effective model training, reducing predictive accuracy—particularly when modeling complex nonlinear behaviors. In particular, constructing models capable of accurately reproducing SMA behavior under repeated cyclic loading and unloading requires experimental data that capture a wide range of loading cycles and the progressive structural changes occurring with each subsequent cycle. This need becomes especially critical for accurately representing hysteresis loops under multiple cycles and varying loading frequencies. A review of the scientific literature on machine learning applications for SMA property prediction reveals a significant lack of studies focused on modeling hysteresis behavior under repeated cyclic loading. Predicting deformation under such conditions is essential, as it allows for estimating the amount of energy dissipated during phase transformations between martensite and austenite—a key parameter for many practical SMA applications.

The aim of this study is to develop and evaluate machine learning models for predicting the hysteresis behavior of SMAs, as well as to ensure the interpretability of these models through the application of Explainable Artificial Intelligence (XAI) techniques. The work focuses on improving strain-prediction accuracy and reducing forecasting errors. The integration of XAI enables the analysis of how individual input parameters contribute to the model’s predictions, thereby enhancing model transparency and supporting the physical validity of the results with respect to the underlying phase-transformation mechanisms in SMAs.

## 2. Materials and Methods

### 2.1. Data Collection and Preparation

The experimental data were obtained from low-cycle fatigue tests of a NiTi shape memory alloy wire with a diameter of 1.5 mm and a length of 210 mm. The wire was supplied by Wuxi Xin Xin Glai Steel Trade Co., Ltd. (Wuxi, China). The chemical composition of the material was 55.78% Ni and 44.12% Ti, with a total impurity content of 0.1% (Table 1).

**Figure 3:**
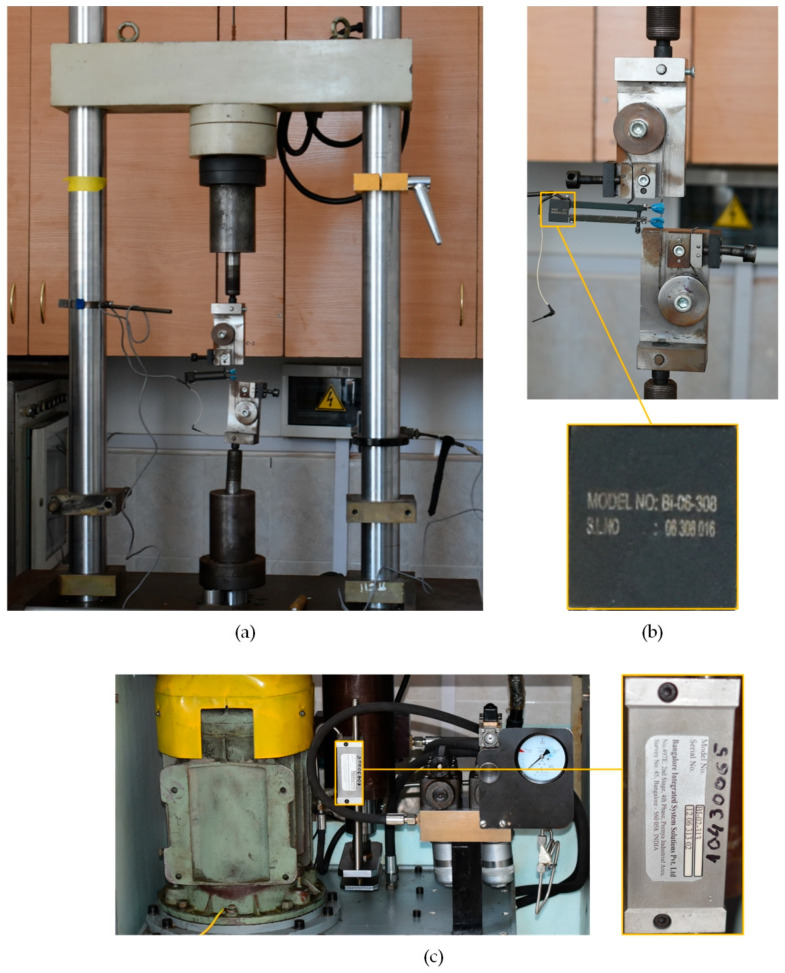
The machine for the experiment: (**a**) general view of the STM-100; (**b**) test sample fixed in the grippers and Bi-06-308 sensor; (**c**) Bi-02-313 sensor.

The Young’s modulus of nitinol in the austenitic state was *E_A_* = 52.7 GPa [62]. The onset of the forward austenite-to-martensite transformation occurred at a stress of σ_AM_ = 338 MPa. The tests were carried out in air at a temperature of 24 ± 1 °C using an STM-100 servo-hydraulic testing machine (Figure 3a) in stress-controlled mode, in accordance with ASTM F2516-14 [63]. Uniaxial tensile fatigue tests were performed under sinusoidal cyclic loading with a stress ratio of 0.1. During testing, the applied force, actuator displacement, and wire elongation were recorded. Experiments were conducted at various loading frequencies (f = 0.1, 0.3, 0.5, 1, 3, 5, and 10 Hz) [64]. Elongation was measured using a Bi-06-308 extensometer (Figure 3b) manufactured by Bangalore Integrated System Solutions (BISS), while displacement was recorded using a Bi-02-313 inductive displacement sensor (Figure 3c).

The relative measurement error of the instruments, as indicated by their calibration certificates, did not exceed 0.1%. Stress σ (MPa) and strain ε (%) were determined from the recorded force–elongation data using the Test Builder software, version 5.3. At least three specimens were tested for each loading frequency. Hysteresis loops were saved for every cycle, which enabled the calculation of instantaneous stress, strain, and the dissipated energy W_dis_, defined as the area enclosed by the loading–unloading loop. In this study, experimentally obtained data from 100 to 250 loading–unloading cycles of the SMA material were used for all seven loading frequencies: 0.1, 0.3, 0.5, 1, 3, 5, and 10 Hz.

### 2.2. Experimental Dataset Description and Correlation Analysis

The experimental dataset consisted of the applied stress σ (MPa), the loading cycle number N, the material strain ε (%), an indicator of loading or unloading, and the loading frequency *f* (Hz). The number of elements in the dataset for each of the seven frequencies is shown in Table 2.

During the preliminary data processing, the dataset was first examined for abnormal values of Stress and Strain that could distort the statistical characteristics or adversely affect model training. To accomplish this, three independent criteria were applied sequentially.

First, the ±3sigma rule, also known as the k-sigma test [65], was applied. For each numerical variable, the mean μ and standard deviation sigma were computed. Any observation in which at least one variable fell outside the interval μ ± 3sigma was considered a potential outlier. This approach relies on the assumption of a quasi-normal distribution. Under a normal distribution, more than 99.7% of all data points are expected to lie within three standard deviations from the mean; therefore, a violation of this boundary indicates atypical behavior in the data. Next, the interquartile range (IQR) method, using the classical multiplier of 1.5, was applied [66]. For each feature, the first (Q_1_) and third (Q_3_) quartiles were computed, followed by the calculation of the interquartile range IQR = Q_3_ − Q_1_. The inner bounds were then defined using the standard formulas Q_1_ − 1.5·IQR and Q_3_ + 1.5·IQR. Any observation that exceeded these limits in at least one variable was flagged as a potential outlier. This method does not rely on any distributional assumptions and is robust to isolated extreme values. To identify local anomalies, a rolling z-score method [67] with a window of 20 points (10 preceding and 10 following the current point) was applied. Within this window, the local mean μ and standard deviation sigma were computed, and the condition |z| > 3 was checked. This approach is capable of detecting short-term spikes or drops that may not affect the global statistics but still disrupt the local structure of the signal. Each criterion operated independently. All candidates for removal were combined, duplicates were eliminated, and the total number of unique suspicious rows was counted. As a result of applying three independent methods for detecting outliers, less than 0.1% of samples were removed from the dataset for each loading frequency, which has no effect on the statistical distribution of the data. Outlier detection at this stage enhanced both the stability and the generalization capability of the models.

A correlation analysis [68] was also performed to evaluate the linear relationships between the input features Stress, Cycle, and UpDown and the output variable Strain, determining the degree of their mutual dependence before constructing machine learning regression models.

**Figure 4:**
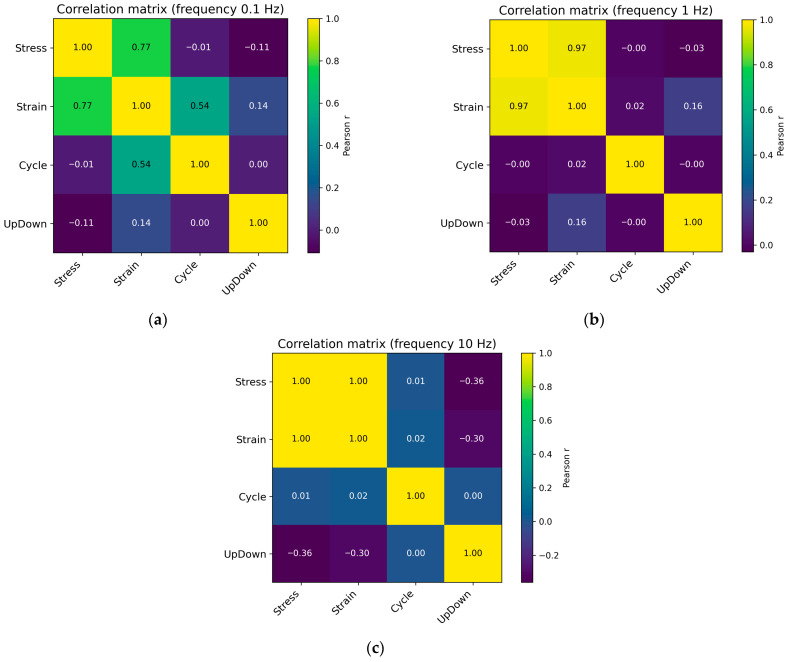
Pearson correlation coefficient matrix for cyclic loading with a frequency of 0.1 Hz (**a**), 1 Hz (**b**), and 10 Hz (**c**).

Figure 4 shows the correlation matrix for load frequencies of 0.1, 1, and 10 Hz.

Similar Pearson correlation matrices were generated for the remaining loading frequencies. As the loading frequency increases, a pronounced strengthening of the positive correlation between stress and strain is observed, accompanied by the disappearance of any meaningful correlation between strain and the cycle number. At the low frequency of 0.1 Hz, the Pearson correlation coefficient for the Stress–Strain pair is approximately 0.77, while Strain still exhibits a noticeable correlation with Cycle (≈0.54), reflecting the accumulation of residual deformation under slow, quasi-static loading conditions. Already at 0.3–0.5 Hz, the Stress–Strain correlation rises sharply to 0.93–0.94, while the Strain–Cycle relationship weakens. Under these conditions, the material has time to unload only partially, and strain becomes almost a direct function of the instantaneous stress rather than of the cycle history. A further increase in frequency (1–5 Hz) elevates the Stress–Strain correlation to 0.97–0.99, whereas all pairs involving Cycle remain close to zero. The mechanical response transitions into an almost elastic regime: the hysteresis loop narrows, and the accumulation effect disappears. At the highest frequency of 10 Hz, the Stress–Strain correlation reaches 1.00. Thus, as the frequency increases, the system evolves from a quasi-static, energy-dissipative regime to a high-rate regime approaching elastic–plastic behavior, in which Stress and Strain are fully correlated, and all other interactions diminish. The pseudoelastic effect becomes substantially diminished [69,70].

Multicollinearity among the input features is practically absent. The pairwise Pearson correlation coefficients in the correlation matrix fluctuate near zero, indicating that no feature pair exhibits a strong linear relationship. According to the commonly accepted threshold of ∣r∣ < 0.3, this confirms the absence of multicollinearity. To additionally verify multidimensional collinearity, the Variance Inflation Factor (VIF) values were computed [71]. All three input features have VIF ≈ 1, which indicates a complete absence of linear dependence between each feature and any linear combination of the remaining features. Although the machine learning models used in this study are not sensitive to multicollinearity, its absence confirms that no informational redundancy exists among the features and serves as an additional indicator of feature set quality.

As part of the data preparation process, the dataset was split into training and test subsets according to the group parameter Cycle, which denotes the loading cycle number of the material. Group-based splitting by Cycle ensured that data from different cycles did not mix between subsets, thereby preventing information leakage and providing an objective evaluation of the model. To construct the subsets, 80% of the cycles were assigned to the training set, while the remaining 20% were allocated to the test set. Thus, the model was trained on cycles within the range 100–250 but evaluated on an independent subset of cycles, ensuring a correct assessment of its generalization ability. Additionally, 10% of the training data was set aside for internal model validation during training. This split was also performed using the group-based principle with respect to the Cycle parameter, guaranteeing that validation cycles did not overlap with the training cycles. This approach enabled an objective assessment of the model’s generalization capability and ensured the reliability of comparisons between the predicted results and the experimental data.

### 2.3. Machine Learning Methods Used in the Study

The study employed neural networks of various architectures, including the Simple Recurrent Neural Network (SimpleRNN), Long Short-Term Memory (LSTM) network, and Gated Recurrent Unit (GRU) network.

### 2.4. Evaluation and Interpretation Methods

In this study, the model’s performance was evaluated using the mean absolute error (*MAE*), mean squared error (*MSE*), coefficient of determination (*R*^2^), and mean absolute percentage error (MAPE) [72].

*MAE* represents the average magnitude of the absolute deviations between the predicted and actual values, allowing one to assess how much the predictions differ from the true values on average. The *MAE* metric was computed using the following formula:

(1) $$MAE=\frac{1}{n}\sum_{i=1}^{n}\left|\varepsilon_{true}^{test}\left(i\right)-\varepsilon_{pred.}^{test}\left(i\right)\right|,$$

where $\varepsilon_{true}^{test}$ is the true value of material strain in the test dataset, $\varepsilon_{pred.}^{test}$ is the predicted value of material strain in the test dataset, and *n* is the size of the test dataset.

*MSE* provides an assessment of the magnitude of the squared error, emphasizing larger deviations because the quadratic function substantially amplifies the impact of large errors. The metric was calculated using the following formula:

(2) $$MSE=\frac{1}{n}\sum_{i=1}^{n}{{(\varepsilon }_{true}^{test}\left(i\right)-\varepsilon_{pred.}^{test}\left(i\right))}^{2}$$

*MAPE* reflects the average percentage deviation of the predicted values from the actual values, making it convenient for interpreting the relative accuracy of the predictions. The metric was calculated using the following formula:

(3) $$MAPE=\frac{1}{n}\sum_{i=1}^{n}\frac{\left|\varepsilon_{true}^{test}\left(i\right)-\varepsilon_{pred.}^{test}\left(i\right)\right|}{\left|\varepsilon_{true}^{test}\left(i\right)\right|}\cdot 100\%$$

The coefficient of determination *R*^2^ indicates how well the model explains the variance of the actual values in the test dataset. It was computed using the following formula:

(4) $$R^{2}=1-\frac{\sum_{i=1}^{n}{\left(\varepsilon_{true}^{test}\left(i\right)-\varepsilon_{pred.}^{test}\left(i\right)\right)}^{2}}{\sum_{i=1}^{n}{\left(\varepsilon_{true}^{test}\left(i\right)-{\bar{\varepsilon }}_{true}^{test}\right)}^{2}}$$

where ${\bar{\varepsilon }}_{true}^{test}$ is the mean value of true material strains in the test dataset.

In this study, the machine learning models were interpreted using the Integrated Gradient (*IG*) method [73,74], which belongs to the class of Explainable Artificial Intelligence (XAI) techniques. Its application made it possible not only to quantitatively assess the contribution of individual features to the model’s decision-making process but also to enhance the transparency and credibility of the obtained results. The Integrated Gradients method computes the contribution of each feature to the model’s prediction by integrating the gradients of the output function along a straight-line path between a baseline (neutral) input and the actual input vector.

Let *F*(*x*) be a differentiable model (e.g., a neural network) that maps the input feature vector *x* = (*x*_1_, *x*_2_, …. *x_n_*) to a scalar prediction. Then, the contribution (integrated gradient) of the *i*-th feature is defined as:

(5) $${IG}_{i}(x)=\left(x_{i}-x_{i}^{\prime }\right)\int_{\alpha =0}^{1}\frac{\partial F\left(x^{\prime }+\alpha \left(x-x^{\prime }\right)\right)}{\partial x_{i}}d\alpha$$

where *x*′ = (*x*_1_′, *x*_2_′, …, *x_n_*′) is the baseline (neutral) input vector, for which the model output *F*(*x*′) is typically close to zero or otherwise non-informative, *α* ∈ [0, 1] is the parameter that traces the path from the baseline input to the actual example, and $\frac{\partial F}{\partial x_{i}}$ denotes the partial derivative (gradient) of the output function with respect to the *i*-th feature.

The resulting integrated gradients ${IG}_{i}(x)$ provide a quantitative measure of the contribution of each feature to the model’s output. The sum of all feature contributions approximates the difference between the predicted value and the baseline output:

(6) $$\sum_{i=1}^{n}{IG}_{i}(x)\approx F\left(x\right)-F(x^{\prime })$$

The method is based on the axioms of Sensitivity and Implementation Invariance, which ensure its reliability and theoretical soundness [73]. This approach enables the evaluation of the influence of each feature on the model output while maintaining mathematical consistency with the model itself.

In our study, the *IG* method was applied to interpret neural network models (RNN, LSTM, GRU), all of which are differentiable architectures. This allowed us to analyze how variations in the input features affect the predicted output and to identify which parameters have the greatest influence on the model’s decisions. Visualization of the resulting feature contributions enabled the identification of the most significant factors and revealed hidden patterns that were not apparent from metric-based evaluation alone.

Additionally, several visualization tools were employed during the model evaluation process to perform a qualitative assessment of how well the predicted values matched the experimental data. The actual vs. predicted plot enabled an examination of the degree of agreement between the predicted and true values. The residual plot illustrated the distribution of prediction errors (the differences between actual and predicted values) as a function of the test sample index. Constructed hysteresis loops allowed for a visual comparison of the shapes of the experimental and predicted stress–strain curves. These visualizations complemented numerical evaluation metrics, providing a deeper insight into the behavior and reliability of the models.

## 3. Results and Discussion

### 3.1. Results of Neural Networks

#### 3.1.1. Architecture

In this study, recurrent neural networks—SimpleRNN, LSTM, and GRU—were employed. These models belong to the class of deep learning architectures capable of effectively capturing temporal dependencies in sequential data. The architectures were built using the TensorFlow/Keras framework [75] with automated hyperparameter selection based on the Hyperband algorithm, which provides an efficient search for optimal configurations in a wide model space. Each model contains an input layer corresponding to the number of input features (Stress, Cycle, and UpDown), four hidden recurrent LSTM layers with nonlinear tanh activation and full sequence return, and an output layer that generates a deformation prediction for each time step. As part of the hyperparameter optimization procedure, the number of neurons in the hidden recurrent layers was varied in the range 32–256 with a step size of 32. The number of hidden LSTM layers varied from one to four. For each recurrent layer, Dropout coefficients were automatically selected within the range of 0.1–0.5 with a step size of 0.1, allowing for the control of regularization and a reduction in the risk of overfitting.

To ensure stable convergence of the learning process in all architectures, the Adam optimizer with exponential learning rate decay was used, implemented through the ExponentialDecay mechanism, where the initial value of the learning rate parameter was 0.001 or 0.0001, after which an exponential decrease in the learning rate with a coefficient of 0.96 was applied every 1000 steps. The final value was fixed at initial_lr = 0.001 for the final models. This approach ensured an adaptive reduction in the learning rate during training, contributing to a gradual approach to the minimum loss function without sharp fluctuations. In addition, an EarlyStopping mechanism with a patience parameter of 150 was used, which automatically stops training when the validation error stabilizes and restores the weights corresponding to the best model state. The combination of automated hyperparameter selection, adaptive optimization, and early stopping made it possible to increase the stability of the learning process, accelerate convergence, and ensure the high generalization ability of recurrent neural networks. The final configuration of each model was determined by the minimum value of the *MSE* loss function on the validation sample.

The architectural parameters and the results of the automated tuning procedure are summarized in Table 3, Table 4 and Table 5.

#### 3.1.2. Performance Evaluation

Model performance was evaluated using standard regression metrics, including MSE, MAE, R^2^, and MAPE. The results for the three architectures and seven loading frequencies are summarized in Table 6.

Analysis of the results presented in Table 6 shows that all recurrent architectures achieved high accuracy on the test data. For all models, the coefficient of determination exceeds R^2^ = 0.999, indicating an almost perfect agreement between the experimental and predicted strain values.

**Figure 5:**
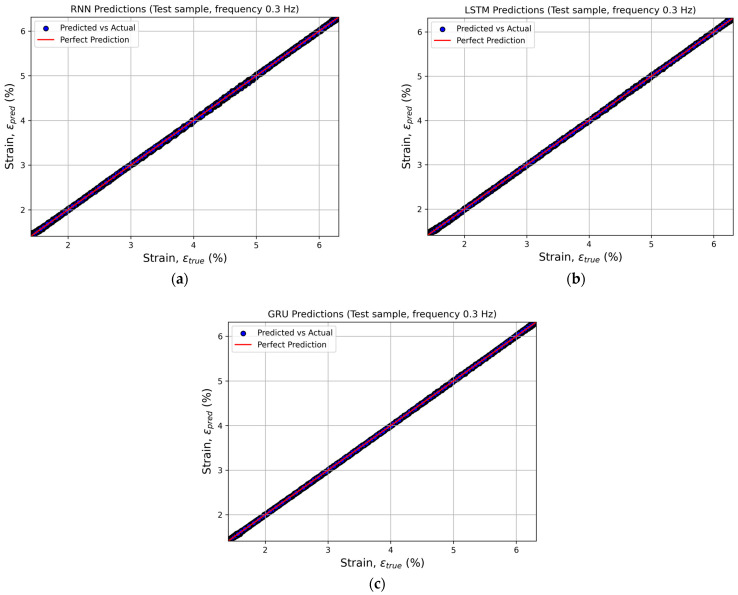
Comparison of the actual and predicted strain values at a loading frequency of 0.3 Hz for recurrent neural networks: (**a**) SimpleRNN, (**b**) LSTM, and (**c**) GRU.

To visually assess the quality of the predictions, actual versus predicted plots were constructed to illustrate the relationship between the experimental and computed strain values. This approach provides an intuitive means of verifying how well the model aligns with the real data. Points located along the line *y* = *x* indicate high prediction accuracy and the absence of systematic bias between the predicted and actual values (Figure 5).

Similar plots were generated for the remaining loading frequencies. The results showed that, for all frequencies, the data points cluster tightly around the bisector of the first coordinate angle.

**Figure 6:**
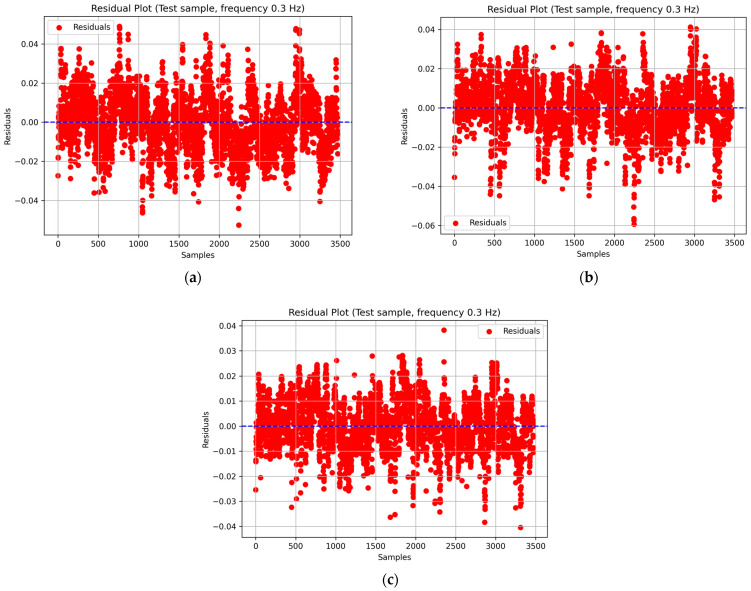
Residual plots for SimpleRNN (**a**), LSTM (**b**), and GRU (**c**) models at a loading frequency of 0.3 Hz.

Additionally, to assess the quality of prediction, residual plots were constructed, reflecting the distribution of differences between actual and predicted deformation values as a function of the measurement number (sample) in the test subsample. This analysis enables the detection of potential systematic errors, local deviations, or hidden trends that may not be apparent from other evaluation metrics (Figure 6).

Similar plots were generated for the remaining loading frequencies. For all models, the residuals exhibit a random distribution with no visible trends or systematic deviations from the zero line, indicating the absence of systematic errors and demonstrating proper model generalization. The amplitude of residual fluctuations does not exceed ±0.05, which confirms the high stability of the predictions and the well-balanced training of the recurrent neural networks.

#### 3.1.3. Verification of Generalization Ability

To evaluate the ability of the developed recurrent neural networks to generalize beyond the training and test ranges, independent testing was performed on loading cycles that were not included in either subset—specifically, cycles 251, 260, 300, 350, 400, 450, and 500. This testing aimed to assess the extrapolation capability of the models, that is, their ability to predict the hysteresis behavior of the alloy as fatigue effects continue to accumulate.

The values of MSE, MAE, R^2^, and MAPE for the three architectures and seven loading frequencies are presented in Appendix A.

The results obtained indicate high prediction accuracy in the area closest to the training sample (251–300 cycles) and a gradual increase in errors with increasing cycle number. The increase in errors during extrapolation to distant cycles (>300) is not a disadvantage of the neural network architecture, but rather a consequence of physically induced material degradation associated with the accumulation of fatigue effects that exceed the information available to the model at the training stage.

At a frequency of 0.1 Hz (Table A1), all models accurately reproduced the hysteresis loops up to cycle 300. Beginning with cycle 350, the accuracy of the SimpleRNN and GRU architectures decreased sharply, whereas the LSTM network maintained acceptable accuracy (R^2^ = 0.9244, MAE ≈ 0.1672 at cycle 400) and demonstrated the strongest extrapolation capability, remaining reliable up to cycle 500. The deformation processes occur more slowly at a frequency of 0.1 Hz, which causes the most pronounced hysteresis behavior of the material. Hysteresis, by its nature, is a process with ‘memory,’ in which the current state of the system is determined by the entire previous load history. With an increase in the number of cycles (after approximately 350), the accumulated error in models that do not work effectively with long-term dependencies becomes critical. The SimpleRNN architecture has the simplest structure and is most susceptible to the vanishing gradient problem, resulting in the loss of information from the early stages of loading in long sequences. Although GRU manages information flows more efficiently using update gate and reset gate mechanisms, its simplified structure with a combined hidden state and memory limits its ability to predict long-term material degradation. In contrast, LSTM is specifically designed to model long-term dependencies through the presence of a separate cell state, which ensures stable information transfer over time. The forget gate mechanism enables the selective rejection of irrelevant data while preserving key features of load evolution over hundreds of cycles, which is crucial for accurately predicting hysteresis loops under low-frequency loading.

At a frequency of 0.3 Hz (Table A2), all architectures preserved high prediction accuracy across the entire range. The LSTM model exhibited the lowest errors and proved to be the optimal architecture for this frequency. At a frequency of 0.5 Hz (Table A3), the prediction accuracy remained consistently high up to cycle 450. At cycle 500, the LSTM model demonstrated the best performance (R^2^ = 0.9912, MAE = 0.0572). Thus, LSTM is the most effective architecture for this frequency. At 1 Hz (Table A4), all models showed excellent agreement with the experimental data up to cycle 400. With further increases in cycle number, the accuracy decreased slightly; however, LSTM maintained the highest performance (R^2^ = 0.9976, MAE = 0.0282 at cycle 500). At a frequency of 3 Hz (Table A5), all models exhibited high prediction quality; however, LSTM produced the lowest errors and the most stable convergence (R^2^ = 0.9867, MAE = 0.0629 at cycle 500). The GRU model exhibited similar accuracy but showed slightly greater variability in error at higher cycle numbers. Therefore, LSTM is the most suitable architecture for a 3 Hz signal. For the 5 Hz frequency (Table A6), the GRU model achieved the best performance (R^2^ = 0.9805, MAE = 0.0614 at cycle 500). At the highest frequency of 10 Hz (Table A7), all models maintained high accuracy within the first extrapolated cycles (251–350), although accuracy decreased for more distant cycles. The LSTM model demonstrated the most stable performance (R^2^ = 0.9979, MAE = 0.0153, MAPE = 0.8% at cycle 500).

Summarizing the results across all frequencies, the following conclusions can be drawn:

- LSTM demonstrated the highest stability and extrapolation accuracy for frequencies of 0.1–3 Hz and 10 Hz;
- GRU proved to be the optimal architecture for 5 Hz;
- SimpleRNN provides acceptable accuracy only within the training range but loses consistency during long-range extrapolation (beyond approximately 350 cycles).

Thus, the LSTM and GRU models demonstrated strong generalization capability and robust extrapolation performance in predicting the hysteresis behavior of SMAs under cyclic loading, making them suitable for long-term forecasting applications.

#### 3.1.4. Comparison of Experimental and Predicted Hysteresis Loops

For all loading frequencies and for all recurrent neural network architectures, experimental and predicted hysteresis loops were constructed for cycles 251, 260, 300, 350, 400, 450, 500, and 600. This graphical representation enabled the visual comparison of the predicted loop shapes with the experimental data, allowing for an assessment of the models’ ability to accurately reproduce phase transitions and fatigue-accumulation effects. Across all frequencies, a high degree of agreement was observed between the predicted and experimental curves, further confirming the physical credibility of the obtained predictions.

**Figure 7:**
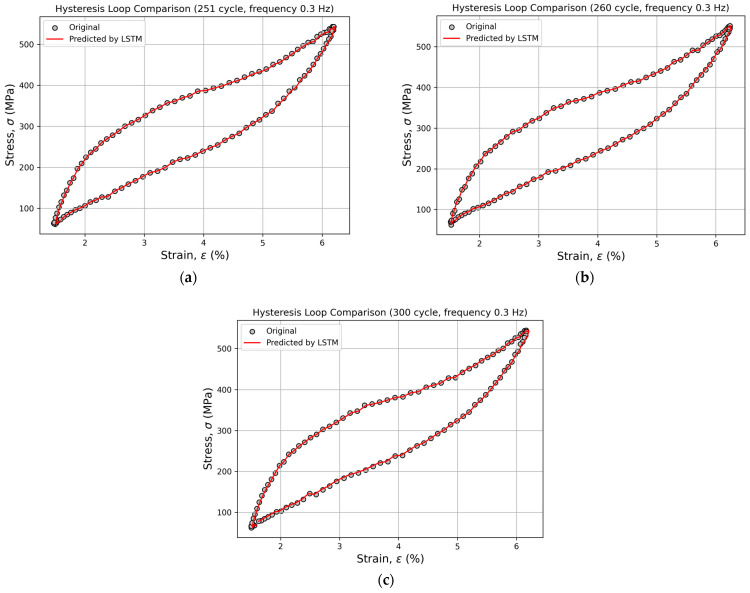
Comparison of experimental and predicted hysteresis loops for cycles 251 (**a**), 260 (**b**), and 300 (**c**) at a loading frequency of 0.3 Hz, obtained using the LSTM model.

**Figure 8:**
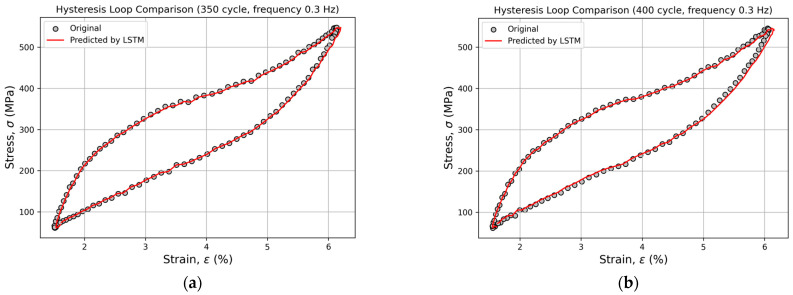

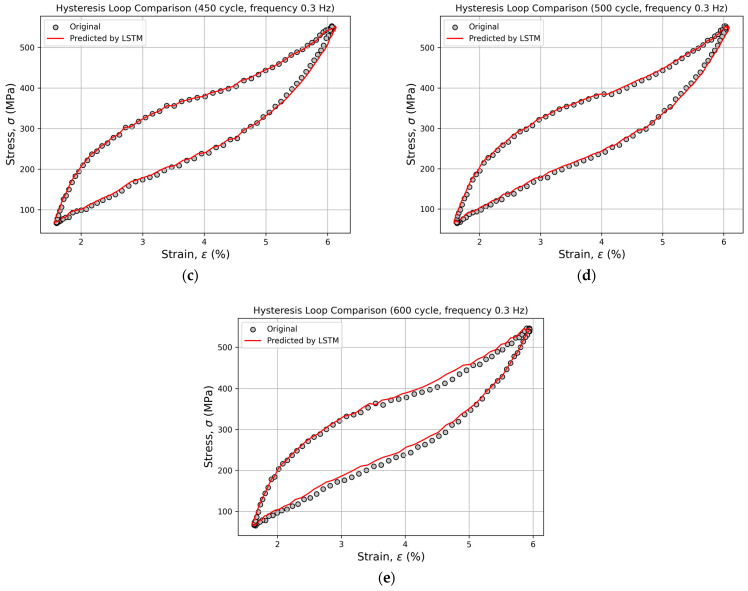
Comparison of experimental and predicted hysteresis loops for cycles 350 (**a**), 400 (**b**), 450 (**c**), 500 (**d**), and 600 (**e**) at a loading frequency of 0.3 Hz, obtained using the LSTM model.

Figure 7 and Figure 8 present an example of the comparison between the experimental and predicted hysteresis loops generated by the LSTM model for cycles 251, 260, 300, 350, 400, 450, 500, and 600 at a loading frequency of 0.3 Hz. Within the range of cycles 251–300, the predicted and experimental curves almost perfectly overlap, demonstrating the model’s high accuracy in reproducing the material’s hysteresis behavior (Figure 7).

Starting from cycle 350, slight deviations become noticeable in the upper portion of the loop (Figure 8).

Even at cycle 600, the model adequately reproduces the phase transitions and the overall loop shape, demonstrating its robust extrapolation capability and strong physical consistency.

### 3.2. Interpretation of Neural Network Results

In this study, the Integrated Gradients method was applied to analyze the SimpleRNN, LSTM, and GRU models. The method made it possible to determine which input parameters (stress (σ), loading cycle number (N), and the loading–unloading indicator (UpDown)) have the greatest influence on the predicted strain (ε).

The analysis was performed at both the local level (for individual loading cycles) and the global level (for the entire dataset). To generalize the results across the full dataset, the mean absolute attribution value was used, representing the average magnitude of the influence of each feature on the model output (attribution). This metric reflects the average contribution of each feature to a single prediction, eliminating dependence on the number of points per cycle or the dataset size. Such an approach ensures a correct comparison of global feature importance across different loading frequencies and models and aligns with standard practices for evaluating global attributions in contemporary XAI research. For an individual cycle, the total importance was computed as the sum of the absolute attribution values for each feature within that cycle. This metric reflects the integrated influence of a feature on the predicted strain across the entire loading–unloading cycle. The use of total importance is particularly appropriate for analyzing the evolution of the influence of physical parameters over time, i.e., as the number of loading–unloading cycles increases.

**Figure 9:**
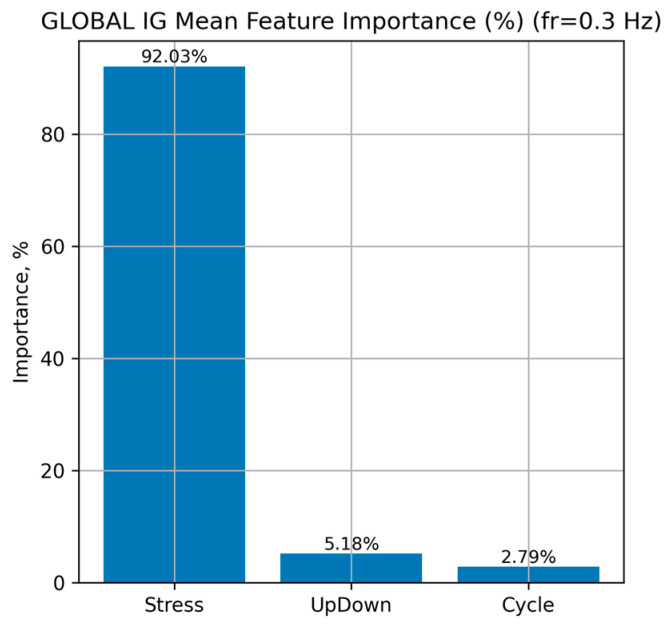
Global distribution of input-feature importance for the LSTM neural network at a loading frequency of 0.3 Hz.

Figure 9 presents the global distribution of feature importance for the LSTM model, obtained using the IG method at a loading frequency of 0.3 Hz

The results show that the dominant factor determining the predicted Strain is Stress, which accounts for approximately 92.0% of the overall mean importance. The features UpDown (approximately 5.2%) and Cycle (approximately 2.8%) contribute substantially less, indicating their secondary influence compared with Stress.

**Figure 10:**
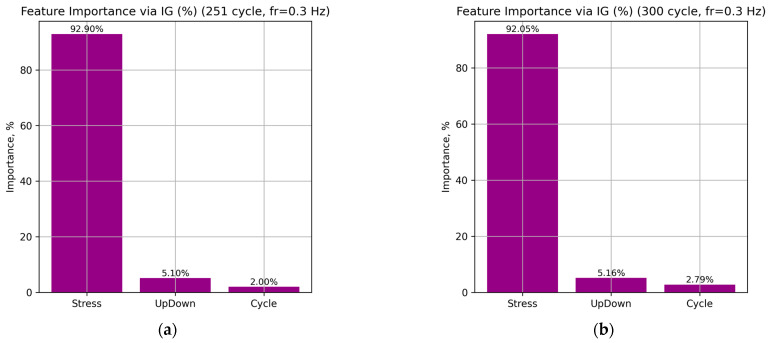

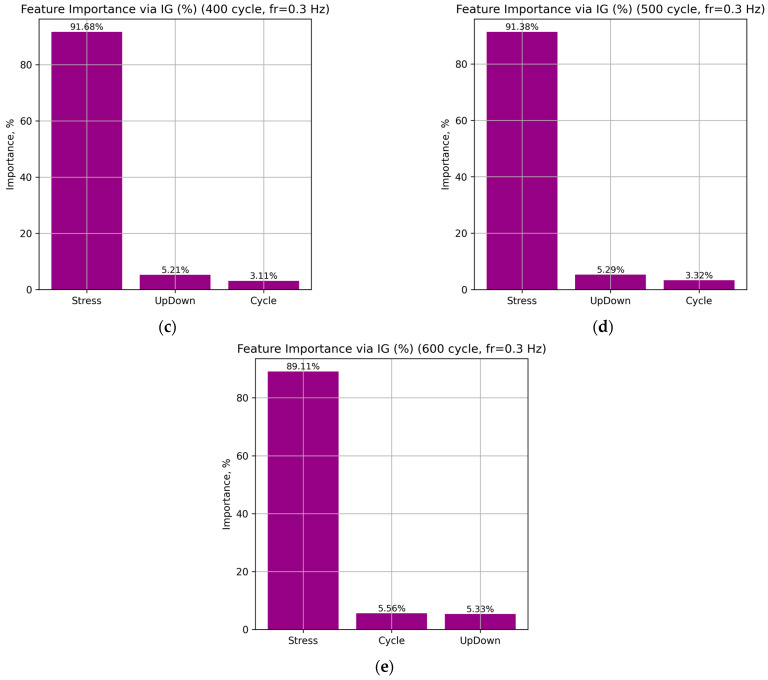
Distribution of the total importance of input features for the LSTM neural network at a frequency of 0.3 Hz: cycles 251 (**a**), 300 (**b**), 400 (**c**), 500 (**d**), and 600 (**e**).

Figure 10 presents the distribution of local feature importance for individual loading cycles (251, 300, 400, 500, and 600) at a frequency of 0.3 Hz for the LSTM neural network.

According to the obtained results, the Stress feature dominates in all cases, accounting for 92.90–89.11% of the total attribution sum, which indicates its leading role in determining the predicted strain. The UpDown feature provides a stable but secondary contribution (5.10–5.33%), associated with transitions between the loading and unloading phases. Meanwhile, the influence of the Cycle parameter gradually increases from 2.00% to 5.56% as the number of cycles increases, indicating that the model correctly captures the accumulation of fatigue effects in the material.

This distribution of attributions is consistent with the physical nature of deformation processes in NiTi SMA. Variations in Stress directly govern the phase transitions between martensite and austenite. The relatively constant contribution of UpDown reflects the model’s consistent reproduction of hysteresis behavior during loading and unloading. The Cycle parameter represents the long-term fatigue accumulation effect.

## 4. Conclusions

This work presents an approach to predicting the hysteresis behavior of NiTi shape memory alloys which integrates recurrent neural networks (SimpleRNN, LSTM, GRU) with XAI-based interpretability methods. The experimental dataset was constructed from loading–unloading cycles at seven loading frequencies (0.1, 0.3, 0.5, 1, 3, 5, and 10 Hz) between 100 and 250 cycles. Generalization capability was evaluated on independent cycles 251, 260, 300, 350, 400, 450, and 500. The data were split according to the Cycle group parameter, which prevents information leakage between subsets and ensures an objective evaluation of model performance.

The results demonstrate high modeling accuracy across the entire frequency range. On the test subsets, all architectures achieved an R^2^ value greater than 0.999, with very low MAE, MSE, and MAPE values. The residuals exhibit a random distribution without systematic trends, confirming proper model training and strong agreement between predictions and experimental measurements. Extrapolation tests showed that high accuracy is retained for cycles 251–300, with errors gradually increasing for later cycles due to the accumulation of fatigue effects. Among the architectures, the LSTM network showed the highest accuracy and strongest extrapolation capability for frequencies ranging from 0.1 to 3 Hz and 10 Hz, whereas the GRU model performed best at 5 Hz. Visual comparison of the experimental and predicted hysteresis loops confirmed that the phase transitions between austenite and martensite were accurately reproduced, even at distant cycles.

Model explainability using Integrated Gradients revealed that Stress is the dominant factor determining the predicted strain. The UpDown feature has a stable but secondary contribution associated with the transition between loading and unloading phases. The influence of the Cycle parameter increases gradually with cycle number, indicating that the model correctly captures the progressive accumulation of fatigue effects in the material. These interpretability results enhance trust in the predictions and support the physical validity of the models.

Future work will focus on integrating Temporal Convolutional Networks (TCNs) as an alternative architecture. The application of TCNs represents a promising direction for further enhancing the model’s extrapolation capability.

## Figures and Tables

**Table 1:** Chemical composition of nitinol (%).

Co	Cu	Cr	Fe	Nb	C	H	O	N
0.005	0.005	0.005	0.012	0.005	0.032	0.001	0.040	0.001

**Table 2:** Distribution of the number of elements in the dataset by cyclic load frequencies.

**Frequency, Hz**	0.1	0.3	0.5	1	3	5	10
**Number of** **elements**	15,251	16,912	3051	16,006	18,573	14,949	5587

**Table 3:** Main hyperparameters of the SimpleRNN-based neural network models.

Frequency, Hz	Neurons per Layer	Dropout
0.1	96-256-224-32	0.2-0.1-0.5-0.3
0.3	192-128-224-32	0.1-0.1-0.5-0.3
0.5	128-64-256-32	0.3-0.1-0.1-0.5
1	128-224-160-128	0.1-0.3-0.2-0.5
3	256-96-192-64	0.1-0.1-0.4-0.2
5	32-256-128-160	0.1-0.2-0.3-0.4
10	256-128-192-64	0.1-0.3-0.4-0.5

**Table 4:** Main hyperparameters of the LSTM-based neural network models.

Frequency, Hz	Neurons per Layer	Dropout
0.1	160-256-128-256	0.2-0.3-0.1-0.3
0.3	224-128-64-256	0.2-0.2-0.1-0.2
0.5	64-256-96-64	0.2-0.1-0.5-0.5
1	256-160-224-64	0.1-0.1-0.5-0.4
3	256-224-32-256	0.3-0.1-0.4-0.2
5	224-224-224-160	0.3-0.2-0.1-0.2
10	224-128-160-96	0.1-0.2-0.5-0.5

**Table 5:** Main hyperparameters of the GRU-based neural network models.

Frequency, Hz	Neurons per Layer	Dropout
0.1	224-256-224-64	0.2-0.5-0.3-0.2
0.3	96-256-32-192	0.1-0.1-0.3-0.4
0.5	32-224-128-64	0.2-0.1-0.3-0.2
1	256-224-128-224	0.1-0.2-0.1-0.1
3	256-224-32-64	0.2-0.2-0.1-0.1
5	160-128-256-160	0.3-0.1-0.1-0.5
10	224-256-224-64	0.5-0.2-0.2-0.3

**Table 6:** Accuracy metrics of the SimpleRNN, LSTM, and GRU models for different loading frequencies.

Frequency, Hz	Model	MSE	MAE	R^2^	MAPE
0.1	SimpleRNN	0.0008	0.0224	0.9996	0.0028
	LSTM	0.0014	0.0276	0.9994	0.0037
	GRU	0.0004	0.0157	0.9998	0.0020
0.3	SimpleRNN	0.0002	0.0112	0.9999	0.0036
	LSTM	0.0002	0.0103	0.9999	0.0037
	GRU	0.0001	0.0080	0.9999	0.0025
0.5	SimpleRNN	0.0003	0.0127	0.9996	0.0044
	LSTM	0.0001	0.0099	0.9997	0.0035
	GRU	0.0002	0.0112	0.9996	0.0039
1	SimpleRNN	0.0007	0.0067	0.9998	0.0029
	LSTM	0.0004	0.0049	0.9999	0.0021
	GRU	0.0004	0.0054	0.9999	0.0023
3	SimpleRNN	0.0007	0.0065	0.9998	0.0032
	LSTM	0.0004	0.0052	0.9998	0.0025
	GRU	0.0003	0.0043	0.9999	0.0021
5	SimpleRNN	0.0004	0.0049	0.9997	0.0028
	LSTM	0.0004	0.0051	0.9997	0.0028
	GRU	0.0002	0.0040	0.9998	0.0023
10	SimpleRNN	0.0007	0.0066	0.9991	0.0036
	LSTM	0.0004	0.0054	0.9994	0.0029
	GRU	0.0005	0.0053	0.9994	0.0029

## Data Availability

The raw data supporting the conclusions of this article will be made available by the authors on request.

## Appendix A

**Table A1:** Accuracy metrics of the SimpleRNN, LSTM, and GRU models at a loading frequency of 0.1 Hz.

Model	Cycle	MSE	MAE	R^2^	MAPE
SimpleRNN	251	0.0011	0.0266	0.9990	0.0029
	260	0.0018	0.0324	0.9982	0.0036
	300	0.0562	0.1823	0.9343	0.0204
	350	0.3457	0.4423	0.5341	0.0496
	400	0.6182	0.6096	0.1619	0.0685
	450	0.6629	0.6303	0.0053	0.0699
	500	0.7200	0.6597	−0.1014	0.0731
LSTM	251	0.0006	0.0223	0.9994	0.0024
	260	0.0007	0.0224	0.9993	0.0024
	300	0.0048	0.0593	0.9944	0.0063
	350	0.0162	0.0966	0.9782	0.0105
	400	0.0557	0.1672	0.9244	0.0188
	450	0.0775	0.1966	0.8836	0.0219
	500	0.1329	0.2663	0.7966	0.0296
GRU	251	0.0008	0.0242	0.9993	0.0026
	260	0.0035	0.0535	0.9967	0.0056
	300	0.0628	0.2319	0.9266	0.0238
	350	0.2212	0.4571	0.7019	0.0474
	400	0.4344	0.6508	0.4110	0.0682
	450	0.6044	0.7665	0.0930	0.0797
	500	0.7636	0.8510	-0.1681	0.0886

**Table A2:** Accuracy metrics of the SimpleRNN, LSTM, and GRU models at a loading frequency of 0.3 Hz.

Model	Cycle	MSE	MAE	R^2^	MAPE
SimpleRNN	251	0.0005	0.0180	0.9998	0.0049
	260	0.0001	0.0097	0.9999	0.0030
	300	0.0007	0.0207	0.9997	0.0056
	350	0.0044	0.0555	0.9983	0.0146
	400	0.0080	0.0738	0.9968	0.0180
	450	0.0089	0.0795	0.9964	0.0197
	500	0.0141	0.1016	0.9941	0.0260
LSTM	251	0.0003	0.0138	0.9999	0.0043
	260	0.0008	0.0077	0.9999	0.0025
	300	0.0003	0.0153	0.9999	0.0052
	350	0.0010	0.0278	0.9996	0.0078
	400	0.0019	0.0368	0.9992	0.0096
	450	0.0018	0.0366	0.9992	0.0113
	500	0.0022	0.0430	0.9990	0.0145
	600	0.0122	0.0838	0.9946	0.0241
GRU	251	0.0001	0.0091	0.9999	0.0024
	260	0.0009	0.0064	0.9999	0.0018
	300	0.0004	0.0152	0.9998	0.0045
	350	0.0010	0.0254	0.9996	0.0060
	400	0.0023	0.0405	0.9990	0.0105
	450	0.0015	0.0327	0.9993	0.0106
	500	0.0028	0.0467	0.9988	0.0151

**Table A3:** Accuracy metrics of the SimpleRNN, LSTM, and GRU models at a loading frequency of 0.5 Hz.

Model	Cycle	MSE	MAE	R^2^	MAPE
SimpleRNN	251	0.0001	0.0130	0.9996	0.0041
	260	0.0002	0.0075	0.9998	0.0027
	300	0.0003	0.0132	0.9994	0.0045
	350	0.0003	0.0152	0.9992	0.0049
	400	0.0006	0.0204	0.9987	0.0067
	450	0.0012	0.0305	0.9973	0.0096
	500	0.0059	0.0682	0.9874	0.0200
LSTM	251	0.0001	0.0074	0.9999	0.0025
	260	0.0001	0.0097	0.9998	0.0032
	300	0.0002	0.0118	0.9994	0.0042
	350	0.0009	0.0271	0.9980	0.0086
	400	0.0008	0.0246	0.9980	0.0086
	450	0.0015	0.0337	0.9966	0.0112
	500	0.0041	0.0572	0.9912	0.0180
GRU	251	0.0001	0.0073	0.9998	0.0028
	260	0.0003	0.0147	0.9995	0.0045
	300	0.0007	0.0234	0.9986	0.0076
	350	0.0012	0.0309	0.9974	0.0103
	400	0.0023	0.0419	0.9949	0.0124
	450	0.0040	0.0567	0.9911	0.0170
	500	0.0094	0.0864	0.9797	0.0250

**Table A4:** Accuracy metrics of the SimpleRNN, LSTM, and GRU models at a loading frequency of 1 Hz.

Model	Cycle	MSE	MAE	R^2^	MAPE
SimpleRNN	251	0.0001	0.0094	0.9997	0.0037
	260	0.0001	0.0117	0.9996	0.0047
	300	0.0005	0.0185	0.9989	0.0087
	350	0.0019	0.0354	0.9959	0.0166
	400	0.0041	0.0546	0.9916	0.0247
	450	0.0076	0.0783	0.9842	0.0337
	500	0.0125	0.1015	0.9735	0.0408
LSTM	251	0.0001	0.0084	0.9998	0.0036
	260	0.0001	0.0077	0.9998	0.0031
	300	0.0001	0.0081	0.9998	0.0034
	350	0.0003	0.0144	0.9993	0.0064
	400	0.0005	0.0198	0.9990	0.0083
	450	0.0008	0.0247	0.9983	0.0108
	500	0.0011	0.0282	0.9976	0.0121
GRU	251	0.0001	0.0081	0.9998	0.0036
	260	0.0001	0.0090	0.9997	0.0039
	300	0.0002	0.0121	0.9995	0.0056
	350	0.0008	0.0210	0.9984	0.0099
	400	0.0013	0.0250	0.9973	0.0123
	450	0.0023	0.0322	0.9953	0.0159
	500	0.0027	0.0368	0.9942	0.0177

**Table A5:** Accuracy metrics of the SimpleRNN, LSTM, and GRU models at a loading frequency of 3 Hz.

Model	Cycle	MSE	MAE	R^2^	MAPE
SimpleRNN	251	0.0001	0.0084	0.9997	0.0044
	260	0.0001	0.0080	0.9997	0.0041
	300	0.0004	0.0155	0.9990	0.0069
	350	0.0015	0.0307	0.9967	0.0139
	400	0.0045	0.0516	0.9909	0.0221
	450	0.0083	0.0693	0.9835	0.0298
	500	0.0114	0.0827	0.9775	0.0367
LSTM	251	0.0001	0.0054	0.9999	0.0030
	260	0.0001	0.0076	0.9998	0.0036
	300	0.0002	0.0107	0.9994	0.0059
	350	0.0006	0.0171	0.9987	0.0084
	400	0.0025	0.0372	0.9949	0.0168
	450	0.0047	0.0506	0.9906	0.0223
	500	0.0067	0.0629	0.9867	0.0290
GRU	251	0.0001	0.0054	0.9999	0.0027
	260	0.0001	0.0043	0.9999	0.0019
	300	0.0002	0.0142	0.9994	0.0074
	350	0.0007	0.0232	0.9985	0.0119
	400	0.0028	0.0492	0.9943	0.0250
	450	0.0049	0.0645	0.9902	0.0329
	500	0.0070	0.0722	0.9862	0.0376

**Table A6:** Accuracy metrics of the SimpleRNN, LSTM, and GRU models at a loading frequency of 5 Hz.

Model	Cycle	MSE	MAE	R^2^	MAPE
SimpleRNN	251	0.0001	0.0061	0.9997	0.0033
	260	0.0001	0.0105	0.9992	0.0055
	300	0.0056	0.0691	0.9797	0.0467
	350	0.0071	0.0731	0.9765	0.0488
	400	0.0106	0.0915	0.9679	0.0579
	450	0.0182	0.1125	0.9479	0.0602
	500	0.0329	0.1492	0.9111	0.0740
LSTM	251	0.0001	0.0054	0.9998	0.0030
	260	0.0001	0.0089	0.9995	0.0046
	300	0.0055	0.0699	0.9799	0.0470
	350	0.0082	0.0860	0.9730	0.0562
	400	0.0112	0.0962	0.9661	0.0642
	450	0.0117	0.0934	0.9664	0.0606
	500	0.0181	0.1137	0.9510	0.0739
GRU	251	0.0001	0.0032	0.9999	0.0019
	260	0.0002	0.0126	0.9991	0.0069
	300	0.0072	0.0815	0.9739	0.0535
	350	0.0063	0.0746	0.9792	0.0493
	400	0.0070	0.0729	0.9788	0.0481
	450	0.0057	0.0578	0.9835	0.0355
	500	0.0072	0.0614	0.9805	0.0367

**Table A7:** Accuracy metrics of the SimpleRNN, LSTM, and GRU models at a loading frequency of 10 Hz.

Model	Cycle	MSE	MAE	R^2^	MAPE
SimpleRNN	251	0.0001	0.0058	0.9995	0.0031
	260	0.0001	0.0076	0.9991	0.0041
	300	0.0003	0.0137	0.9973	0.0069
	350	0.0002	0.0103	0.9987	0.0055
	400	0.0005	0.0185	0.9959	0.0089
	450	0.0006	0.0182	0.9951	0.0090
	500	0.0014	0.0335	0.9902	0.0181
LSTM	251	0.0001	0.0056	0.9996	0.0029
	260	0.0001	0.0041	0.9997	0.0022
	300	0.0001	0.0107	0.9984	0.0058
	350	0.0002	0.0120	0.9981	0.0061
	400	0.0001	0.0100	0.9988	0.0054
	450	0.0003	0.0159	0.9971	0.0083
	500	0.0003	0.0153	0.9979	0.0082
GRU	251	0.0001	0.0055	0.9996	0.0030
	260	0.0001	0.0053	0.9995	0.0029
	300	0.0003	0.0125	0.9974	0.0064
	350	0.0004	0.0149	0.9969	0.0083
	400	0.0016	0.0310	0.9874	0.0158
	450	0.0058	0.0576	0.9565	0.0298
	500	0.0099	0.0836	0.9302	0.0456
